# Association of the imaging characteristics of desmoplasia on digital breast tomosynthesis and the Ki-67 proliferation index in invasive breast cancer

**DOI:** 10.3325/cmj.2021.62.59

**Published:** 2021-02

**Authors:** Kristina Samaržija, Zoran Jurjević

**Affiliations:** Department of Radiology, Karlovac General Hospital, Karlovac, Croatia

## Abstract

**Aim:**

To evaluate the imaging characteristics of desmoplasia on digital breast tomosynthesis (DBT) and their association with the Ki-67 index.

**Methods:**

Seventy-seven malignant spiculated breast masses were analyzed in terms of tumor size, length and width of spicules, coverage of tumor margin with spicules, and the number of spicules. The Ki-67 index was obtained from surgically removed tumor specimens.

**Results:**

The average spicule length was significantly negatively associated with a high Ki-67 (*P* = 0.005, odds ratio [OR] 0.252, 95% confidence interval [CI] 0.094–0.676), ie, the lesions with longer spicules had a 3.968 times lower odds of having a high Ki-67 than the lesions with shorter spicules. The average spicule width at the base was significantly positively associated with Ki-67 (*P* = 0.004, OR 3.939, 95% CI 1.520–10.209), ie, the lesions with thick spicules had a 3.939 times higher odds of having a high Ki-67 than the lesions with thin spicules. The lesions with more than 20 spicules and those with partially spiculated margins more frequently had a high Ki-67 than those with fewer spicules and fully spiculated margins, but the differences were not significant.

**Conclusion** The spiculation analysis could be used as a non-invasive method providing information about malignant lesions. The tumor proliferative activity, and therefore the patient's prognosis, might be predicted before biopsy directly from DBT images.

One of the essential processes in tumor progression is the invasion of the surrounding healthy tissues by a malignant neoplasm (1-3). In many epithelial neoplasms, such as breast cancer, this process includes the tumor-associated fibrotic reaction of the host tissues, known as desmoplasia (3-5). In the last two decades, the analysis of tumor progression began to include the study of tumor microenvironment, especially changes in stromal collagen. Provenzano et al noticed three different types of collagen morphology at the tumor-stromal interface, introducing the term tumor-associated collagen signatures (TACS) (6). The first type develops during tumor formation, when cancer-associated fibroblasts overproduce collagen, but it retains a wavy appearance (TACS-1). As the tumor progresses, collagen fibers stretch and align, distributing themselves parallel to the tumor boundary (TACS-2). In the final phase, multiple collagen fibers form bundles and reorient themselves perpendicularly to the tumor (TACS-3) (6). These collagen fibers are used by the cancer cells as tracks to migrate away from the primary tumor, so the regions containing TACS-3 correspond to the sites of focal invasion into the stroma (6-11). This type of stromal collagen reorganization at the tumor-stromal boundary, ie, desmoplasia, is responsible for the spiculated appearance of invasive breast cancer on mammography (12).

Uncontrolled proliferation, as one of the key characteristics of malignancy (13,14), is most commonly estimated with the Ki-67 proliferation index (14). Ki-67 is a nuclear non-histone protein present in all active phases of proliferating cell cycle (15). This protein is an excellent marker of the cancer growth fraction and, consequently, biological aggressiveness of the tumor (16). This index is defined as the percentage of cells with Ki-67-positive nuclear immunostaining among the total number of malignant cells (14). Numerous studies demonstrated the association of a high Ki-67 with worse prognosis and confirmed it as a reliable marker for disease outcome (13,17,18).

One of the most significant breast imaging techniques developed in the last few decades is digital breast tomosynthesis (DBT) (19). This tomographic technique allows multiple low-dose exposures to be obtained as the x-ray tube rotates along a limited arc around the breast. In this way, thin parallel slices are generated throughout the breast, similar to those obtained in other three-dimensional techniques (19). The method reduces the masking effects of breast tissue overlap, thus overcoming an important limitation of standard mammography (19). The addition of tomosynthesis to 2D mammography has improved cancer detection and decreased false-positive rates (20,21). Furthermore, removing the effects of tissue overlap has made lesion margins more visible and improved lesion characterization (22-24).

No study so far has examined the individual features of spicules in relation to disease outcome. Most literature data on the morphological features of spiculated lesions are descriptive, since spicule measurement is not a part of routine clinical practice. Therefore, we aimed to evaluate the imaging characteristics of desmoplasia in malignant spiculated breast masses using the benefits of tomosynthesis in the assessment of lesion margins, and to assess their association with high Ki-67 index, a poor-prognosis marker. The association between these two parameters would indicate that this non-invasive imaging method could predict biological behavior of cancers and patient prognosis.

## PATIENTS AND METHODS

This retrospective single-center study was performed in the Radiology Department of the Karlovac General Hospital, the largest hospital in Karlovac County, serving approximately 100 000 residents. The population of Karlovac County is older than the Croatian average, with 25% of women over 65 years of age. The incidence of breast cancer is slightly above the national average, with breast cancer being the most common cancer type among women. In our department, about 5000 mammograms are performed annually.

The study involved all patients who presented to our department with spiculated breast masses observed on DBT and who were histopathologically confirmed to have invasive cancers in the period between May 2015 and November 2018. Inclusion criteria were clear visibility of spiculated lesion borders in DBT slices and histopathologically confirmed malignancy with available Ki-67 index. There were 70 patients, with 77 lesions (6 patients had multifocal or bilateral lesions). Since no biological difference relevant to the study was expected between unifocal and multifocal or bilateral lesions, we analyzed all lesions as one group. The patients were women aged between 40 and 86 years (mean age, 67 years). Pathological diagnoses were established from surgically excised specimens, and Ki-67 index was available for all lesions. The study was approved by the Ethics Committee of Karlovac General Hospital.

DBT examination was performed on a Hologic Selenia Dimensions 3D Mammography system (Marlborough, MA, USA). The standard examination technique was used, ie, tomosynthesis in both the craniocaudal and mediolateral oblique projection with synthesized 2D images, auto-filter exposure model (system selects filter, kV and mAs), and full compression mode (up to 100 Newtons). Digitally stored images were analyzed on a Hologic SecurView DX workstation (Single 12 Megapixel LCD Barco display), with the regions of interest selected manually.

**Figure 1:**
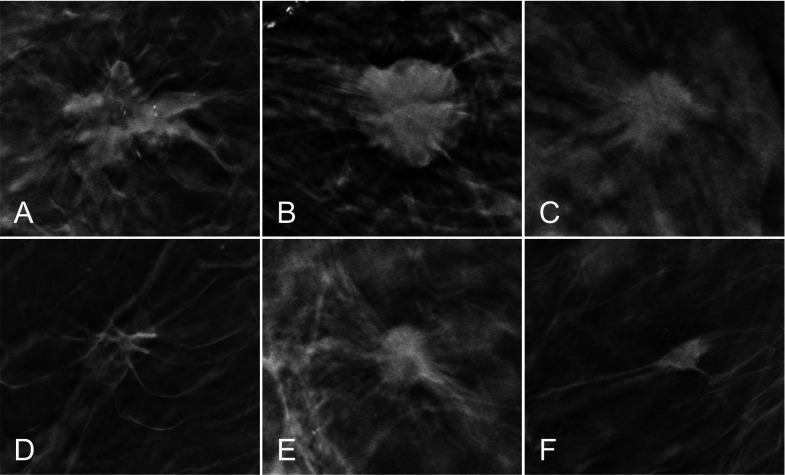
(**A**) A fully spiculated lesion with 5-20 shorter thick spicules – high Ki-67; (**B**) A fully spiculated lesion with >20 shorter thin spicules – high Ki-67; (**C**) A fully spiculated lesion with >20 shorter thick spicules – high Ki-67; (**D**) A fully spiculated lesion with 5-20 longer thin spicules – low Ki-67; (**E**) A fully spiculated lesion with >20 longer thin spicules – low Ki-67; (**F**) A partially spiculated lesion with <5 longer thin spicules – low Ki-67.

The malignant spiculated breast masses were analyzed in terms of tumor size, length, width, and the number and distribution of spicules around the mass. All parameters were measured at the tomosynthesis slice where the lesion was most clearly visible. The tumor size was measured at the longest diameter of the central tumor mass, excluding the spicules. The length of all spicules was also measured, and the ratio of average spicule length to tumor size was determined. The lesions with the ratio <1 were categorized as having shorter spicules, and the lesions with the ratio ≥1 were categorized as having longer spicules relative to the tumor mass. The spicule width was measured at its base. The spicules with average width at the base <1 mm were considered thin, while those with average width ≥1 mm were considered thick. Because the spicules were not observed around the entire tumor perimeter, the lesions were categorized as fully (more than half of the volume) or partially (less than half of the volume) spiculated according to the coverage of the tumor margin with spicules. The total number of spicules was determined for each lesion, and the lesions were classified into arbitrary groups: those with fewer than 5, 5 to 20, and more than 20 clearly visible spicules (Figure 1).

All measurements were performed independently by two radiologists with respectively 8 and 12 years of experience in mammography reading, 4 of which were in DBT. They had discrepancies n 8 cases when measuring the spicule length, in 14 cases when measuring the spicule width, in 6 cases when measuring the spicule number, and in 7 cases when measuring the spicule distribution. In these cases, both radiologists repeated the measurements, and the disagreements were resolved by consensus.

We assessed the association of all the desmoplastic characteristics with the Ki-67 proliferation index obtained by the immunohistochemical analysis of surgically removed tumor specimens. The resected breast tissue was routinely fixed immediately after surgery in 10% buffered formalin solution and later embedded in paraffin. Paraffin blocks were cut into 4-μm sections, stained with hematoxylin and eosin, and analyzed by pathologists in our institution. The immunohistochemical staining of Ki-67 was performed with appropriate monoclonal mouse antibodies (clone MIB-1, Dako, Glostrup, Denmark) as per the manufacturer’s instructions. When staining was homogenous, cells in five randomly selected fields ( × 40 objective) were counted. In the case of heterogenous staining, counting was done in the hot spots and on the invasive edge of the tumor. At least 1000 tumor cells (five 200-cell fields) indicated cell proliferation. More than 20% positive cells among the total number of tumor cells pointed to high Ki-67 expression, whereas less than 20% pointed to low Ki-67 expression (25-27).

### Statistical analysis

Normality of data distribution was tested with the Kolmogorov-Smirnov test. The association between the parameters of spiculated breast masses and high Ki-67 was assessed with the Pearson chi square test or the Fisher-Freeman-Halton exact test as appropriate. The odds ratio (OR) and 95% confidence intervals (CI) were calculated to quantify the association strength. We also constructed a receiver operating characteristic (ROC) curve to determine the parameters differentiating high from low Ki-67 expression and calculated the area under the curve (AUC) with 95% CI. *P* values <0.05 were considered significant. Statistical analysis was performed with MedCalc Statistical Software, version 19.4.0 (MedCalc Software Ltd, Ostend, Belgium, 2020).

## RESULTS

Between May 2015 and November 2018, a total of 77 DBT-detected spiculated breast lesions were histopathologically confirmed as invasive breast cancer. Most of them (n = 50, 64.9%) were found in 61-80-year-old patients. A total of 46.8% (n = 36) of lesions had low Ki-67 expression. Most lesions (n = 41, 53.2%) were 10-20 mm in diameter (Table 1). Patient age or lesion diameter were not associated with Ki-67 level.

**Table 1:** Patients' and lesions' characteristics (77 lesions)

Characteristic	Number (%) of lesions
**Patient’s age (years)**	
≤50	3 (3.9)
51-60	16 (20.8)
61-70	27 (35)
71-80	23 (29.9)
>80	8 (10.4)
**Ki-67 expression**	
high Ki-67	41 (53.2)
low Ki-67	36 (46.8)
**The diameter of the lesion (mm)**	
<10	19 (24.7)
10-20	41 (53.2)
>20	17 (22.1)
**The length of spicules**	
longer	28 (36.4)
shorter	49 (63.6)
**The width of spicules**	
thick	37 (48.1)
thin	40 (51.9)
**The number of spicules**	
<5	3 (3.9)
5-20	30 (39)
>20	44 (57.1)
**Distribution of spicules**	
fully spiculated	58 (75.3)
partially spiculated	19 (24.7)

Out of 77 spiculated lesions, 28 (36.4%) had longer spicules and 49 (63.6%) had shorter spicules relative to size of the tumor mass. The lesions with longer spicules were more frequently associated with a low Ki-67 (19/28, 67.9%), whereas the lesions with shorter spicules were more frequently associated with a high Ki-67 (32/49, 65.3%). The association between the spicule length and high Ki-67 was significant (*P* = 0.005, OR 0.252, 95% CI 0.094-0.676). Consequently, longer spicules were negatively associated with a high Ki-67 (OR<1), ie, the lesions with longer spicules had 3.968 times lower odds of having a high proliferation index than the lesions with shorter spicules.

Considering the spicule width, 40 (51.9%) lesions at their base had thin spicules and 37 (48.1%) had thick spicules. The lesions with thick spicules were predominantly (26/37, 70.3%) associated with a high Ki-67, whereas the lesions with thin spicules were predominantly associated with a low Ki-67 (25/40, 62.5%). The association between the spicule width and high Ki-67 was significant (*P* = 0.004, OR 3.939, 95% CI 1.520-10.209). Accordingly, thick spicules were positively associated with a high Ki-67 (OR>1), ie, the lesions with thick spicules at the base had a 3.939 times higher odds of having a high Ki-67 than the lesions with thin spicules.

According to the number of spicules, 3 (3.9%) lesions had fewer than 5, 30 (39%) had between 5 and 20, and 44 (57.1%) had more than 20 spicules. High Ki-67 was more often found in the lesions with more than 20 spicules (27/44, 61.4%), but the association was not significant (*P* = 0.263).

Regarding the coverage of tumor margin with spicules, 58 (75.3%) lesions were fully spiculated and 19 (24.7%) were partially spiculated. Partially spiculated lesions more frequently than fully spiculated lesions had a high Ki-67 (11/19, 57.9% vs 30/58, 51.7%), but the association was not significant (*P* = 0.640) (Table 2).

**Table 2:** The association of patients' age and morphological characteristics of lesions with Ki-67

	High Ki-67- number (%)	Low Ki-67- number (%)	P	Odds ratio
**Patient age (years)**			0.583†	
≤50	2 (66.7)	1 (33.3)		
51-60	7 (43.8)	9 (56.2)		
61-70	14 (51.9)	13 (48.1)		
71-80	15 (65.2)	8 (34.8)		
>80	3 (37.5)	5 (62.5)		
**The lesion diameter (mm)**			0.219*	
<10	7 (36.8)	12 (63.2)		
10-20	25 (61)	16 (39)		
>20	9 (52.9)	8 (47.1)		
**The length of spicules**			0.005*	0.252
longer	9 (32.1)	19 (67.9)		
shorter	32 (65.3)	17 (34,7)		
**The width of spicules**			0.004*	3.939
thick	26 (70.3)	11 (29.7)		
thin	15 (37.5)	25 (62.5)		
**The number of spicules**			0.263†	
<5	1 (33.3)	2 (66.7)		
5-20	13 (43.3)	17 (56.7)		
>20	27 (61.4)	17 (38.6)		
**The distribution of spicules**			0.640*	
fully spiculated	30 (51.7)	28 (48.3)		
partially spiculated	11 (57.9)	8 (42.1)		

*Pearson chi square test.

†Fisher-Freeman-Halton exact test.

**Figure 2:**
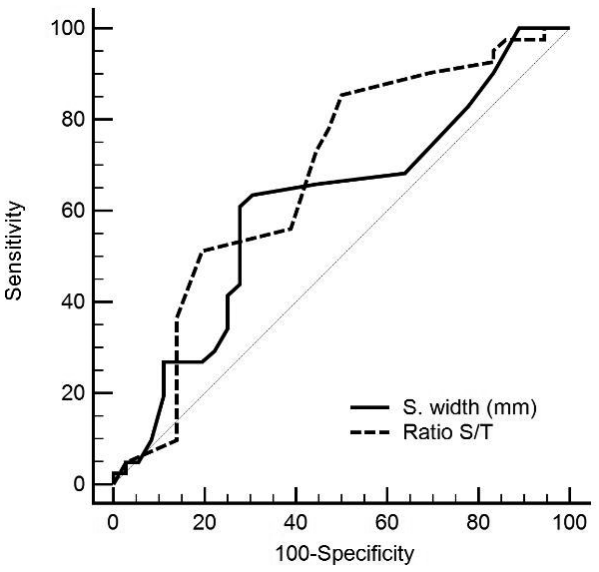
Receiver operating characteristic curves for the parameters differentiating between low and high Ki-67 expression lesions on digital breast tomosynthesis. Ratio S/*t* = the ratio of average spicule length to tumor size.

High and low Ki-67 expression lesions could be distinguished based on spicule/tumor ratio (AUC = 0.679, 95% CI 0.562-0.780, *P* =0.005). At the optimal cut-off value for spicule/tumor ratio of ≤1.1, the sensitivity was 85.37% and the specificity was 50.00%. For the spicule width, AUC was 0.613 (95% CI 0.496-0.722, *P* = 0.084, Figure 2).

## DISCUSSION

In this study, two physical characteristics of spicules were associated with the Ki-67 level. The average spicule length was significantly negatively associated with a high Ki-67, ie, the lesions with longer spicules relative to central tumor mass more frequently had low proliferation index. Malignant lesions develop spiculated margins as a result of changes in stromal collagen at the tumor-stromal interface. It is logical to assume that slow growing lesions, with low proliferation index, will generate a progressive stromal response leading to the formation of long spicules. On the other hand, rapidly evolving cancers, with a high proliferation index, do not have enough time for an extensive desmoplastic reaction, which results in shorter spicules. Sannomiya et al showed that breast cancers with well-defined margins, which did not cause a stromal reaction, had a greater proliferative capacity and therefore grew by pushing the surrounding tissues (29). Other researchers have reported that spiculated tumors were strongly associated with a lower Ki-67 (30,31). As the spicules grow together with the tumor mass, the spicule length and the size of tumor mass are interrelated. Therefore, we used the average spicule length relative to the size of tumor mass (longer or shorter than the mass), because we considered it to be a more conclusive parameter than an absolute spicule length.

In contrast to the average spicule length, the association between the average spicule width and Ki-67 was significantly positive, ie, the lesions with thick spicules at the base were significantly more likely to have a high proliferation index. The central tumor mass histologically consists of tumor stroma, which contains different proportions of tumor cells. Spicules develop from connective tissue as overproduced collagen at the tumor/stromal boundary orients itself perpendicular to the tumor border (TACS 3). Since these collagen fibers create a pathway enabling cancer cells to invade the environment, besides connective tissue cells, spicules may also contain tumor cells. Because the focal invasion begins at the point where the spicule meets the mass, tumor cells are expected to be more abundantly present at the spicule base than at its end. This is why we measured the spicule width at their base. Berment et al similarly found that very fine spicules observed on mammography were generally related to a fibrous reaction, whereas short and thick spicules probably followed the infiltration of tumor cells into the connective tissue (32). Therefore, the spicules that are thick at the base and rich in tumor cells were expected to be positively associated with a high proliferation index.

A spiculated lesion is the most typical mammographic manifestation of breast cancer, and a spiculated margin is a feature with highest positive predictive value for malignancy (32-34). A tumor presenting as a spiculated mass is more likely to be invasive than cancer *in situ* (32). The appearance of spiculated masses varies greatly depending on the thickness, length, and number and distribution of spicules around the mass (32). Despite this, no study so far has examined the individual features of spicules in relation to disease outcome. Most studies have provided descriptive data on the morphological features of spiculated lesions, since spicule measurement is not a part of routine clinical practice. Sampat et al, by measuring the physical characteristics of spiculated masses on mammography, demonstrated that the spicule parameters can be reliably measured within the level of variability typical for other radiological measurements (35). Their primary problem, however, was not to measure a spicule after it is located but to differentiate spicules from the summation of the shadows of normal fibroglandular tissue. We measured the morphological features of spicules at the central tomosynthesis slice where the lesion was most clearly visible, which eliminated the major weakness of conventional mammography caused by tissue superimposition with structures out of plane. Our study showed a good to very good inter-observer agreement, meaning that performing such measurements is possible for clinical purposes.

In the last decade, methods for extracting quantitative features from medical images have been developed; this practice is termed radiomics (36). In 2019, Tagliafico et al investigated whether quantitative radiomic features extracted from DBT were associated with Ki-67 expression in breast cancer (37). They identified five features that differentiated patients with low from those with high Ki-67 expression, concluding that it might be possible to estimate the activity of the tumor directly from DBT images. Despite the differences between their and our study, they also did not find features with AUC higher than 0.7. Therefore, they believed that radiomic features on DBT did not strongly discriminate between low and high Ki-67-expressing breast cancers and considered it necessary to validate their findings in larger studies.

In this study, Ki-67 proliferation index was estimated by manual cell counting. It is a time-consuming process as at least 500–1000 cells have to be counted (14). In addition, high variability has been reported among pathology laboratories (38-40). Although digital image analysis (DIA) has emerged as an alternative to manual scoring, potentially offering a standardized diagnostic solution for Ki-67 assessment in breast cancer, high concordance between manual scoring and DIA has been reported (41,42). Madabhushi et al reviewed the challenges and opportunities in digital pathology as a new source of “big data.” They believe that advances in computer imaging might transform pathology from a qualitative to a quantitative discipline, a process in which it was far behind radiology (43). Digital pathology could serve as a bridge to radiology, enabling the identification of radiologic image markers associated with molecular changes and allowing better disease characterization (43).

One of the limitations of this study is a relatively small number of lesions, so the frequency of some variables is low, which may reduce the power of the statistical analysis and limit the validity of our conclusions. The association of the number and distribution of spicules with Ki-67 did not reach statistical significance in discriminating low from high Ki-67 expression lesions, and the P value in the ROC curve analysis for the spicule width was close to the significance level, probably due to insufficient power of the study. This indicates a need for further studies with a larger sample size and a larger number of analyzed lesions, which would allow a combination of features to form potential subgroups for further analysis. This limitation may be attributed to a single-center experience, so further research should encompass several different institutions. Second, we used the proliferation index as a surrogate for outcomes rather than cancer survival, which could not be used since less than 5 years has passed since the diagnosis.

As a conclusion, our study suggests that the analysis of spiculation could serve as a non-invasive method to provide additional information about malignant lesions. We found that spicule length and width discriminated between low from high Ki-67 expression lesions. All this indicates that DBT images might be used before biopsy to directly predict the tumor proliferative activity, and therefore, the patient prognosis.
